# Electromagnetic Fields and Free Radicals

**DOI:** 10.1289/ehp.112-a726a

**Published:** 2004-09

**Authors:** Richard G. Stevens

**Affiliations:** University of Connecticut Health Center, Farmington, Connecticut, E-mail: bugs@neuron.uchc.edu

The article “Magnetic-Field–Induced DNA Strand Breaks in Brain Cells of the Rat” by Lai and Singh (2004) is interesting. The possibility that exposure to anthropogenic nonionizing radiation and/or electromagnetic fields (EMFs) might increase oxidative potential and free radical burden in cells may be a unifying theme for possible adverse biological consequences. Two articles published in *EHP* in the past explored two ideas in this regard. In the first article, we (Stevens and Kalkwarf 1990) pointed out *a*) that ferritin has a stable magnetic moment of 3.8 Bohr magnetons, and *b*) that on the basis of reports from Bawin and Adey (1976) and others that EMFs could alter calcium homeostasis, increases in free radicals could be expected. In the second article, I postulated specifically that “EMF-induced loss of iron from its intracellular storage protein, ferritin, might increase oxidative stress” (Stevens 1993).

This is an intriguing area of inquiry at the scientific level that may also have health implications.
